# Developing a medication safety self-assessment tool for high-alert medications in community pharmacies

**DOI:** 10.1016/j.rcsop.2025.100664

**Published:** 2025-10-01

**Authors:** Rositsa Koleva, Anita Währn, Ercan Celikkayalar, Sonja Kallio, Raisa Laaksonen

**Affiliations:** a Clinical Pharmacy Group, Department of Pharmacology and Pharmacotherapy, Faculty of Pharmacy, University of Helsinki, Viikinkaari 5 E, PL 56, 00014 Helsinki, Finland; b Rauma 1^st^ Pharmacy, Rauma, Finland; c Lahti 2^nd^ Pharmacy, Lahti, Finland; d Wellbeing Services County of Kanta-Häme, Hämeenlinna, Finland; e Association of Finnish Pharmacies, Helsinki, Finland

**Keywords:** High-alert medication, Medication safety, Self-assessment tool, Community pharmacy, Risk assessment, Risk management

## Abstract

**Background:**

High-alert medications are recognised as those carrying heightened risk of causing significant patient harm when used erroneously.

**Objective:**

To develop a high-alert medications self-assessment tool for Finnish community pharmacies.

**Methods:**

The tool was developed using a three-phase Delphi method and is based on the Institute for Safe Medication Practices´ Medication Safety Self Assessment® for High-Alert Medications, which comprises 380 items. A pre-Delphi round was first conducted to assess tool's applicability for Finnish pharmacies, followed by two Delphi rounds with a multidisciplinary expert panel evaluating the applicability and desirability of each item. A consensus rate of 70 % was defined. Following the Delphi rounds, the tool was finalized through refinement, removal of duplicates, and reorganization.

**Results:**

Consensus was reached on 114 items, resulting in a finalized self-assessment tool organized into eight sections covering medicine groups such as insulin and oral diabetes medicines, anticoagulants, opioids, immunosuppressants, methotrexate, and over-the-counter high-alert medications. After the first Delphi round, 33 items were accepted without changes and 97 were revised. After the second Delphi round, 77 items were transferred to the final tool as such, 35 were modified and 21 were removed.

**Conclusion:**

The developed high-alert self-assessment tool offers a structured method for evaluating existing practices and implementing targeted safety measures, addressing a specific need in community pharmacies, where such resources are limited. While further validation and implementation research are needed, the tool represents a practical step toward enhancing medication safety and promoting continuous improvement in pharmacy practice.

## Highlights

• The self-assessment tool helps community pharmacies identify and manage high-alert medications, improving patient safety.
• Developed through a Delphi process, the tool is reliable, evidence-based resource for risk assessment and mitigation.
• The tool provides practical guidelines and best practices supporting continuous quality improvement and patient-centered care.
• It offers a structured, systematic approach for identifying risks and implementing targeted safety measures.
• The tool aligns with international medication safety priorities and offers a model adaptable to other healthcare systems.

## 1 Introduction

High-alert medications are medications that carry a heightened risk of causing significant harm when used in error.^1^ They do not necessarily cause more side effects than others, but when an error occurs, the consequences are more likely to be severe due to factors as a narrow therapeutic index, potential for severe interactions, or specific handling requirements.^2^^,^^3^ Both the World Health Organization (WHO) and the Institute for Safe Medication Practices (ISMP) identify high-alert medications as a leading cause of preventable harm globally.^4^^,^^5^ To prevent medication errors and minimize harm, specific safety measures should be established and implemented.^2^^,^^5^^,^^6^

In primary care, common high-alert medication groups include immunosuppressants, opioids, insulins, chemotherapeutic agents, and antithrombotic agents.^7^ In community pharmacies, over-the-counter painkillers, potassium chloride, and medications requiring additional pharmaceutical counselling are also recognised as high-alert medications.^8^^,^^9^ These are frequently consumed; in 2023, they were among the ten most used over-the-counter medications in Finland, with a total consumption of 93.9 DDD/1000 inhabitants/day.^10^ In 2022, several high-alert medications, such as metformin, paracetamol, ibuprofen, oxycodone, and buprenorphine, were among the ten most frequently reported in Finnish pharmacy error reports.^11^ Between 2013 and 2014, over one-third of medication error-related injuries compensated by the Finnish Patient Insurance Centre were associated with high-alert medications.^12^

Look-alike, sound-alike (LASA) medications on the other hand, are medications that might be confused due to orthographic (look-alike) and phonetic (sound-alike) similarities.^13^ Errors involving LASA medications can occur at any stage of the medication-use process, and may cause serious adverse events particularly when involving high-alert medications.^14^ In 2013, nearly 20 % of reported dispensing errors involved these medications, with LASA medications being a leading contributing factor both in Finland and internationally.^15^, ^16^, ^17^ Additionally, more than 26 % of documented medication-related problems in Finnish pharmacies involved high-alert over-the-counter medications, particularly non-steroidal anti-inflammatory drugs (NSAIDs).^18^ Common issues included incorrect indication, overuse, drug-drug interactions, and duplicated medication. Similar findings from another study highlighted frequent overuse and challenges with administration of over-the-counter medications.^19^

International studies on medication safety in community pharmacies vary in definitions and contexts, making comparisons difficult. Research on high-alert medications in these settings are limited and often focused toward hospital pharmacies.^20^^,^^21^ Studies on self-assessment tools also highlight a lack of resources tailored to community pharmacy needs.^22^^,^^23^ Existing tools such as the ISMP Medication Safety Self Assessment® for Community/Ambulatory Pharmacy and the Agency for Healthcare research and Quality (AHRQ) Community Pharmacy Safety Culture Survey provide general guidance but do not directly address the risks of high-alert medications in community pharmacy practice such as over-the-counter medications and the high volume of direct client interactions.^24^^,^^25^ Findings from Spain and Australia show that high-alert medications such as antithrombotic agents, anti-inflammatory medicines, opioids and medicines used to treat diabetes - are frequently involved in errors in community pharmacies.^7^^,^^26^^,^^27^ LASA medicines are another major contributor, ranking as the second most common cause of errors.^28^^,^^29^ Despite differences across studies, the importance of identifying errors, fostering blame-free reporting culture, and providing staff training remains consistently emphasized^16^^,^^30^^,^^31^.

In Finland, community pharmacies play a crucial role in promoting medication safety and managing outpatient risks.^32^, ^33^, ^34^ The need for tools supporting the safe use of high-alert medications has been recognised.^35^ In 2017, a list of high-alert over-the-counter medications alongside with a checklist for identifying high-risk patients were developed.^8^ In 2019, a medication safety self-assessment tool for community pharmacies was introduced and later updated and more recently, in 2023, the Finnish Medicines Agency (Fimea) launched a National High-Risk Medicines Classification to improve medication safety across healthcare settings.^9^^,^^36^^,^^37^ While these tools assist in identifying high-alert medications and associated risks, there remains a clear need for a comprehensive self-assessment tool tailored specifically to community pharmacies.

## Aims

The aim of this study was to develop a self-assessment tool for identifying high-alert medications and supporting their safe use in community pharmacies.

## 2 Methods and materials

**Fig. 1:**
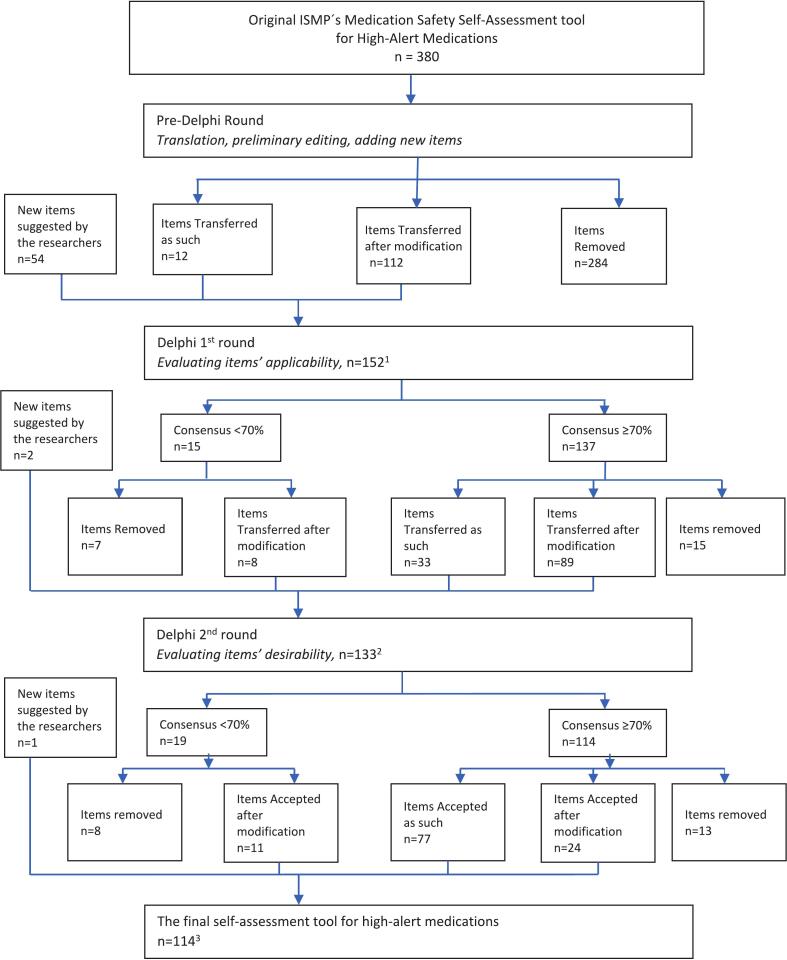
The stages of the Delphi study to develop a self-assessment tool for high-alert medications in community pharmacies.^1^ During the preliminary phase two items were divided into two separate items.^2^ One item was moved from Section 3 to Section 1; three items were divided into separate items and three items were merged with another item.^3^ One new item was added by merging parts of 2 items.

The self-assessment tool for identifying high-alert medications was developed using the Delphi consensus method. The development of the tool was based on Medication Safety Self-Assessment® for High-Alert Medications, including 380 assessment items in 12 sections with the permission of the Institute.^38^ The ISMP tool was chosen for the study due to its comprehensiveness and the organization's extensive experience with high-alert medications and safety measures. The development consisted of three phases: 1) pre-Delphi round, followed by preliminary editing and translating the tool into Finnish; 2) use of the Delphi method in two rounds; and 3) final refining of the tool (Fig. 1).

### 2.1 The Delphi method

The Delphi is a technique that seeks to obtain consensus on the opinions of experts through a series of structured questionnaires called rounds.^39^ The strengths of the method are anonymity, iteration, and controlled feedback.^40^ During the Delphi process, different perspectives, hypotheses, and arguments for the solution of the problem are produced, making it well suited for developing evidence-based recommendations for healthcare environments.^40^^,^^41^ It has been utilised to modify existing self-assessment tools for use in Finnish community pharmacies and healthcare.^9^^,^^22^^,^^36^^,^^43^ In Delphi studies, the consensus rate is usually predetermined typically between 75 and 85 %.^44^ For this study, a 70 % consensus threshold was set, corresponding to a clear majority of respondents and aligning with previous similar studies.^9^^,^^22^^,^^43^ Experts anonymously rated the items of the self-assessment tool in one pre-Delphi round and two Delphi rounds. Based on experts´ responses and comments received to the electronic questionnaires, the final self-assessment tool was developed.

### 2.2 Pre-Delphi phase and initial modification of the self-assessment tool

As part of the pre-Delphi round, the applicability of ISMP's original tool developed for health systems, containing 380 items, for use in community pharmacies was assessed. The expert panel (*n* = 18) included pharmacists participating in a High-Alert Medicines course in the Community and Hospital Pharmacy Specialisation Programme, University of Helsinki, in 2019. These pharmacists had experience with medication safety, high-alert medications, and had work experience in Finnish community pharmacies. Participation was confidential and panellists gave consent for the use of their data in this study.

The main researchers (RK and AW) independently reviewed the items that all expert panellists of the pre-Delphi round had found unsuitable for community pharmacies. If both researchers agreed on the omission of an item, it was removed. The remaining items were translated into Finnish and, both clarifying and substantive changes were made to the items to better reflect Finnish pharmacy practice.

After the pre-Delphi round and initial modifications, 152 items remained in the self-assessment tool. Half of the original 12 sections were deleted resulting in the removal of 75 % of the original items. The most common reasons for removing items were: use or dispense of medicines in hospital environment (44 %); relevance to IV administration (14 %); relevance to monitoring occurring in hospital settings (11 %); and prescribing-related items (11 %).

Because the original tool was developed for use in hospitals and health care facilities, it lacked some high-alert medication groups relevant to community pharmacies, such as oral diabetes medications, over-the-counter medications and those requiring additional pharmaceutical counselling.^38^ These items (*n* = 54) were added to the tool based on international and national recommendations and guidelines by the researchers (RK and AW).^7^ Two other researchers (EC and SK) reviewed the Finnish translation of the items and suggesting modifications some of the items, the addition of new items, as well as the removal of duplicated items or items that were not applicable to pharmacies. The researchers (RK and AW) then made the final modifications to the items improving the clarity of the self-assessment tool by rearranging the sections and renaming some of them.

### 2.3 Selection of experts

Medication safety experts with a background in pharmacy (*n* = 30), medicine (*n* = 10) and nursing (*n* = 7) were identified for the multidisciplinary expert panel. Of these, 44 experts were selected by the research team through purposive sampling, and three were suggested through snowball sampling.^44^ Panellists were invited from a wide range of working environments, including expert organizations, regulatory authorities, pharmacies, hospitals, and other healthcare facilities across Finland, to ensure a diverse perspective on the topic. Invitations were sent to 47 experts, and a survey link was provided to 45, as two were unable to participate. Participation was voluntary and anonymous, and the participants could withdraw at any time, and Consent was implied by completing the survey.

### 2.4 Delphi survey

Two Delphi rounds were conducted and the items that reached the predefined consensus (70 %) were selected for the final tool. Before the second round, all panellists received a summary of the results from the first round, allowing them to re-evaluate their responses.

The Delphi survey rounds were conducted electronically via the eDelphi program (Metodix Oy). An e-mail invitation was sent to the expert panel one week before the start of the first Delphi round describing the aim and schedule of the study. On the first day of each Delphi round, a link to the eDelphi survey was sent to the panellists, along with responding instructions and data protection policy, both available on the eDeplhi platform.

In each Delphi round, experts were given five weeks to respond. In addition to research-related topics, they were asked to provide information on their professional background. Throughout both rounds, experts had the opportunity to submit comments, suggest modifications, and evaluate the clarity of the items.

### 2.5 Piloting the Delphi survey

The survey for the first Delphi round was piloted in September 2021 with two pharmacists, who, based on their professional and educational backgrounds, could have qualified for the expert panel. They provided feedback on the clarity and comprehensibility of the instructions, the technical functionality of the eDelphi platform, and the time required to complete the survey. No alterations to the survey were necessary, but clarifications were added to the instructions for responding to avoid misinterpretations. These pilot responses were not included in the research material.

### 2.6 The first Delphi round: data collection and analysis

The first Delphi round took place between October and November 2021. During the five-week response period, weekly reminders were sent, with two in the final week. The expert panellists were asked to assess the applicability of each item of the self-assessment tool for use in community pharmacies at the time of the study, and also approximately in ten years' time in the future, when medication safety work related to high-alert medications could be more developed.

Three response options for each item were used: the item is suitable as such; the item is suitable when modified; and the item is not suitable for the tool. Experts were encouraged to justify or comment on their own responses and also had the opportunity to suggest new items. The experts could change their responses until final submission of the survey. It was also possible to submit responses to some of the sections or some of the items.

In general, when an item received at least 70 % consensus regarding its applicability, and no change proposals for the item were made, the item was carried over to the second Delphi round without modification. Items that received 70 % consensus but also received suggestions for improvement were revised based on the panellists´ feedback before being included in the second round. Item that received less than 70 % consensus were either removed from the tool or modified according on the panellists' suggestions and then transferred to the second round. All decisions were made through joint considerations by three members of the research team (RK, AW, EC).

### 2.7 Implementation and analysis of the second Delphi round and final editing

The second Delphi round took place between April and May 2022; the response time was five weeks. Reminder messages were sent as previously. Expert panellists were given access to anonymous feedback from the first round, showing all responses, comments, and the number of respondents. The purpose of the feedback was to guide experts to justify their own choices and opinions.^39^

In the second round, the panellists were asked to evaluate the desirability of the items, i.e., whether the items were considered necessary for the tool. Three response options were used: the item is desirable as such; the item is desirable when modified; and the item is not desirable for the tool. No new items could be proposed. In the second Delphi round, the panellists not only saw the first round comments, but also second round comments in real time on the electronic eDelphi platform, enabling anonymous dialogue.

Items with at least 70 % consensus in the second round were included in the tool either as such or modified based on the comments. So, the final self-assessment tool includes the items approved both in the first and second rounds. Final modifications were made (RK, AW, EC, RL) to the tool, which considered all comments received during the Delphi rounds. The language of the sections was clarified, any overlapping sections were removed, and their order was made more logical.

### 2.8 Research ethics

In accordance to the research ethics guidelines established by Finnish National Board on Research Integrity and the advice sought from the Research Ethics Committee in Humanities of the University of Helsinki, this kind of research that is based on voluntary, anonymous and confidential participation of non-vulnerable adults in a study on a non-sensitive topic that does not include clinical interventions to patients, does not require seeking a statement from a research ethics committee.^45^ The study was conducted in accordance with the good ethical practices.^45^^,^^46^

## 3 Results

### 3.1 Results of the first Delphi round

Depending on the section of the self-assessment tool, 17–26 experts (response rate 38–58 %) participated in the first Delphi round. In the first round, 81 % (*n* = 21) of the respondents were pharmacists, 11 % (*n* = 3) were nurses and 8 % (n = 2) were physicians.

The experts evaluated a total of 152 items, of which 96 had been in the original tool and modified for the Delphi, representing 25 % of all items in the original tool. In total, 33 items were transferred to the second round as such, while 97 items were modified and two new items were added. In six out of eight sections, modifications were made to at least 64 % of the items (Table 1). The consensus limit (70 %) had not been achieved in the case of 15 items, of which seven were removed. Additionally, another 15 items were removed. These included items related to HIV medicines, which are primarily dispensed in hospital pharmacies, and items concerning emergency medical services and reversal agents, as these are not relevant to community pharmacy practice.

**Table 1:** Results of Delphi round 1 – response rate, number of comments received on self-assessment items and number of modifications made to the items.

Section of the Self-assessment tool, items (n)	Experts participated in evaluating items, n (%)	Comments written by experts, n	Average comments per item, n	Modifications made to items, n	Average modified items in section, %
1. General High-Alert Medications (*n* = 47)	26–23, (58–51 %)	271	5.76	30	64 %
2. Over-the-counter High-Alert Medications and Those Requiring Additional Pharmaceutical Counselling (n = 30)	20, (44 %)	105	3.50	21	70 %
3. Insulin and Oral (non-insulin) Diabetes Medications (*n* = 20)	20–18, (44–40 %)	146	7.30	13	65 %
4. Chemotherapy (*n* = 11)	18, (40 %)	37	3.36	8	73 %
5. Methotrexate for Non-Oncologic Use (n = 7)	18, (40 %)	12	1.71	6	86 %
6.Anticoagulants (*n* = 14)	20–18, (44–40 %)	50	3.57	6	43 %
7.Opioids (*n* = 11)	17, (38 %)	74	6.73	9	82 %
8. Immunosuppressants and Carbamazepine (*n* = 12)	17, (38 %)	20	1.67	4	33 %

The participants wrote a total of 715 comments, averaging 4.7 comments per item. Over one third (38 %) concerned the first section, to which the most changes were made (*n* = 30/97). Comments concerned mainly the need to clarify items, suggestions for additional content, and need to separate or simplify items so that each contained only a single statement. Based on the comments three items were divided into a total of six items improving their clarity, and another three items were merged into other items to avoid duplications. To ensure that some items would be easier to understand, examples were added as suggested by the experts. Changes were also made to the order of the items consistent terminology was applied throughout the tool. No new items were proposed to the tool. Although the experts were asked to evaluate the items with a view to the future, some experts thought that certain items would be impossible to implement in the pharmacies at the time of the study. However, these items achieved the consensus limit and were included in the tool.

Items related to pharmacists possessing knowledge about comprehensive medication review, which were not mandatory at that moment in Finland, were removed. Items related to pharmacists' duties when dispensing any medicine in general rather than dispensing high-alert medicines in particular were also excluded. In addition, overlapping or duplicate items, were removed, as well as those concerning responsibility of a physicians or another healthcare facility.

### 3.2 Results of the second Delphi round and final modifications

The second Delphi round was attended by 9–14 experts (response rate 20–31 %), who evaluated the desirability of the items in the final self-assessment tool. Most participants, 79 % (*n* = 11) were pharmacists, 14 % (*n* = 2) nurses, and 7 % (n = 1) physicians.

The second Delphi round included 133 remaining items. The consensus limit (70 %) was not reached in the case of 19 items. Of these, eight were removed, one was merged with another item, and the remaining 10 were deemed desirable by the research team, and included after modifications. Additionally, 25 items were modified as suggested by the panellists, bringing the total number of modified items to 35 (Table 2). Two new items were added, as they were considered desirable and significant for promoting safe practices. To enhance clarity, elements from two separate items were merged into a new version, with the rest of the items retained as originally presented. Primarily, due to overlaps, 10 items, which had reached consensus, were deleted from the tool, bringing the total number of removed items to 21.

**Table 2:** Development of assessment items from the original ISMP's Self-Assessment Tool through Delphi rounds 1 and 2 to the final Self-Assessment Tool. Items were divided into two, or, to avoid duplications, merged, and clarified.

Original ISMP's Self-Assessment Tool	Delphi round 1	Delphi round 2	Final Self-Assessment Tool for High-Alert Medications
*One item regarding identification of contraindications and drug-interactions.* All inpatient and/or outpatient orders are entered into a computer order entry system and screened electronically against the patient's current medications and medical profile to identify potential contraindications, interactions, and duplicate therapy before high-alert medications are administered, unless a delay in administration could result in patient harm.	*The item is divided into two items, and modified for community pharmacy setting, and new content is developed.* The pharmacy has an integrated interaction program that automatically reports drug interactions with high-alert medications. Contraindications are determined by counselling the patient about his/her illnesses or conditions that might affect the suitability of the medicine. The pharmacy uses patients´ up-to-date medication list in electronic form (National medication list).	*The items are modified for improved clarity and content is deleted.* The pharmacy has an interaction program integrated into the pharmacy system, which automatically reports drug interactions when high-alert medications are dispensed. The pharmacy uses patients´ up-to-date medication list in electronic form (National medication list).	*The items reached consensus.* The pharmacy has an interaction program integrated into the pharmacy system, which automatically reports drug interactions when delivering high-alert medication. The pharmacy uses patients´ up-to-date medication list in electronic form (National medication list).
*Two items regarding expression of names and dosing instructions of medicines.* The abbreviations MSO4 for morphine and MgSO4 for magnesium sulfate (which could be confused with each other), and DTO for deodorized tincture of opium (which could be mistaken as diluted opium tincture), are never used when expressing the drug names in computer order entry systems, order sets, protocols, guidelines, MARs/eMARs, ADC screens, infusion pump screens, drug storage bins, pharmacy labels and/or AUTOMATED SYSTEM LABELS, and any other format used to communicate the drug in the facility. The abbreviation “U” or “u” is never used for units when expressing medication (e.g., heparin, insulin) doses in any paper, label, or electronic format used to communicate medications in the facility.	*The items are modified for community pharmacy setting, and for improved clarity and examples are added.* Abbreviations are not used in medical counselling, pharmacy software and dosing instructions in order to avoid confusion. To avoid misunderstandings, the abbreviations of the dosage instructions are written out in full on the label (e.g. ml = millilitres, IU = units, inj = injection, 1 × 2 = 1 tablet/ capsule/ dose 2 times a day, s.c. = subcutaneous, p.o. = by mouth).	*One item is modified for improved clarity.* Abbreviations are not used in medical counselling, pharmacy software and dosing instructions in order to avoid confusion. To avoid misunderstandings, the abbreviations of the dosage instructions are written out in full to the label and the electronic prescription (e.g. ml = millilitres, IU = units, inj = injection, 1 × 2 = 1 tablet/ capsule/ dose 2 times a day, 2 × 1 = 2 tablets/capsules/doses once a day, s.c. = subcutaneous, p.o. = by mouth).	*The items are merged.* To avoid misunderstandings, the abbreviations used in medical counselling, pharmacy software and dosing instructions are written out in full e.g., ml = millilitres, IU = units, inj = injection, 1 × 2 = 1 tablet/ capsule/ dose 2 times a day, 2 × 1 = 2 tablets/capsules/doses once a day, s.c. = subcutaneous, p.o. = by mouth).

The experts wrote 219 comments, concerning the clarity and exactness of the items, and the consistent use of some terms. These comments were considered, and in the final editing stage all sections were rearranged, and the language was checked. Overall, a significant number of items (*n* = 316) were removed from the original self-assessment tool. Consequently, the final tool contains 64 items (16.8 %) from the original tool, each of which has been modified at least twice. The reasons for removing items during the different stages of the study are shown in Table 3. The final high-alert medications self-assessment tool consists of 114 items (Table 4**, Supplementary material 1**).

**Table 3:** Number of removed items in descending order and reason for their removal during the pre-Delphi phase, Delphi round 1 and Delphi round 2, and examples of removed items.

Categories of reasons for removing items	Removed items				
	Pre-Delphi round⁎, n	Delphi round 1⁎⁎, n	Delphi round 2⁎⁎, n	All Delphi rounds, n (%)	Examples of removed items
Items related to hospital-dispensed medicines including IV administrated medicines	148	4	–	152 (47 %)	“Dantrolene and any required diluent is readily available (within 10 min of diagnosing a malignant hyperthermia event) in all patient care areas where succinylcholine is stocked (even if succinylcholine is only available in emergency intubation supplies).” Original self-assessment tool
Items related to healthcare other than community pharmacy, such as hospital system, processes, treatment or intervention, and patient monitoring	77	9	5	91 (28 %)	“The organization has conducted a thorough assessment to identify all locations where moderate sedation of patients occurs (including outpatient locations, if applicable) to standardize the care, monitor these practice sites, and provide oversight to promote safety.” Original self-assessment tool
Items overlapped with other items	29	5	13	47 (14 %)	“To ensure the safe use of chemotherapy agents, medicine delivery intervals are monitored.” *And* “If the delivery interval of high-alert medication is too short, the dosage and the reason for the premature need for the medication are determined from the customer.” Delphi round 2
Items related to prescribing of a medicine	30	4	3	37 (11 %)	“Standard order sets exist and are used to prescribe adult, pediatric, and/or neonatal electrolyte replacement therapy and include required patient monitoring.” Original self-assessment tool
Altogether	284	22	21	327	

⁎ During the pre-Delphi round, original items were removed from the self-assessment tool.

⁎⁎ During Delphi rounds 1 and 2, both original and modified items were removed from the self-assessment tool.

**Table 4:** Modification of the sections in the original ISMP's self-assessment tool and number of items in each phase of the study.

	ISMP's original MSSA for High-Alert Medications	Delphi round 1	Delphi round 2	Final Self-Assessment Tool
Section	1. General High-Alert Medications (*n* = 33)	1. General **High-Risk Medicines** (n = 47)	1. General **High-Risk Medicines** (*n* = 41)	1. General **High-Alert Medications** (*n* = 36)
	Neuromuscular Blocking agents (*n* = 15)	N/A	N/A	N/A
	Concentrated Electrolytes Injection (*n* = 26)	N/A	N/A	N/A
	Magnesium Sulphate Injection (*n* = 22)	N/A	N/A	N/A
	Moderate Sedation in Adults and Children, Minimal Sedation in Children (*n* = 40)	N/A	N/A	N/A
	N/A	2. Over-the-counter (OTC) High-Alert Medications Those Requiring Additional Pharmaceutical Counselling (n = 30)	2. Over-the-counter (OTC) High-Alert Medications Those Requiring Additional Pharmaceutical Counselling (*n* = 28)	2. Over-the-counter (OTC) High-Alert Medications Those Requiring Additional Pharmaceutical Counselling (*n* = 24)
	3. Insulin, **Subcutaneous and Intravenous** (*n* = 45)	3. Insulin **and Oral (non-insulin) Diabetes Medications** (n = 20)	3. Insulin and Oral (non-insulin) Diabetes Medications (n = 15)	3. Insulin and Oral (non-insulin) Diabetes Medications (*n* = 13)
	4. Chemotherapy, **Oral and Parenteral** (*n* = 48)	4. **Chemotherapy** (n = 11)	4. Chemotherapy, **Cytostatics** (*n* = 9)	4. **Chemotherapy** (*n* = 6)
	5.Methotrexate for Non-Oncologic Use (n = 7)	5. Methotrexate for Non-Oncologic Use (n = 7)	5. Methotrexate for Non-Oncologic Use (*n* = 7)	5. Methotrexate for Non-Oncologic Use (n = 6)
	Lipid-Based Medications and Conventional Counterparts (n = 9)	N/A	N/A	N/A
	6. Anticoagulants (*n* = 43)	6. Anticoagulants(n = 14)	6. Anticoagulants (n = 12)	6. Anticoagulants (*n* = 8)
	7. Opioids (60)	7. Opioids (n = 11)	7. Opioids (n = 13)	7. Opioids (n = 12)
	Neuraxial Opioids and/or Local Anesthetics (*n* = 32)	N/A	N/A	N/A
	N/A	8. Immunosuppressants, **HIV Medicines** and Carbamazepine (n = 12)	8. **Immunosuppressants and Carbamazepine** (n = 8)	8. Immunosuppressants and Carbamazepine (n = 9)
Items, n	380	152	133	114

## 4 Discussion

This study developed a self-assessment tool for use in community pharmacy that promotes medication safety, based on the Medication Safety Self Assessment® for High-Alert Medications.^38^ The tool has been developed as a comprehensive and practical resource to support pharmacists in community pharmacies in accurately identifying high-alert medications within their own inventory and implementing safeguards to ensure their safe handling and use. The development of a new tool was deemed necessary and timely, as no comparable tool was available in Finland, nor could similar instruments be identified in the international literature. Indeed, the need for identification and risk management of high-alert medicines is described in the national Guide to producing a pharmacotherapy plan and Client and Patient Safety Strategy and Implementation Plan.^3^^,^^47^ The tool was developed to meet the needs of Finnish community pharmacies, considering their operating methods and range of high-alert medicines. The assessment items of the self-assessment tool focus on identifying high-alert medications and the primary risks associated with their use, storing, administration, and disposal. The tool can be used to increase the awareness of pharmacists and pharmacy staff about good practices and methods that foster the medication safety of high-alert medications. The tool can be used in the development, planning, implementation, and monitoring of a good and safe high-alert medicines related operating culture.

### 4.1 Modifying the original ISMP tool

Throughout the study, it became more evident that the ISMP tool developed for hospital environment is not directly applicable to community pharmacy practice. The original tool is comprehensive, with the majority of the items focused on medications used or dispensed in hospitals, prescribing practices, procedures or systems specific to hospital environments. Consequently, only a quarter of the original tool's items remained for the expert assessment in the Delphi rounds. In the final version, less than a fifth of the items in the ISMP's tool were retained.

Applying the items of the original tool required knowledge of the operational system of Finnish community pharmacies, the legislation concerning them, the selection of medicines of community pharmacies, the roles, and responsibilities of different professional groups in pharmacies, and the information systems used by pharmacies and their characteristics. Most original assessment items required in-depth modifications, such as adding content to or splitting it into two or more separate items for clarity. The original tool lacked key high-alert medications available in community pharmacies and related practices and policies. These were added to the tool by the research team in the pre-Delphi phase and during Delphi rounds based on the suggestions from the experts and researchers. However, the study showed that it is possible to use an original ISMP tool to develop a versatile medication safety tool for community pharmacies employing the Delphi method. This aligns with previous studies using other ISMP's medication safety tools to develop medication safety tools for use in other healthcare settings through the Delphi method.^22^^,^^36^

### 4.2 Information content of the self-assessment tool

The developed self-assessment tool can be used in community pharmacies to identify high-alert medications and highlight the primary risks associated with their use. The tool considers the high-alert medications available in community pharmacy by classes, as well as the risks associated with their safe handling, storing, dispensing and disposal. The tool also includes medicines whose names and packaging are similar to each other (LASA medicines), and medicines used by high-risk patients such as children, pregnant women, and older adults.

The content of the medication safety tool has been developed describing risks and critical points related to both the medicinal substance and operating methods and practices. The tool also includes various development objects that may not currently be implemented in pharmacies but are seen as desirable and necessary development targets for enhancing medication safety. When using the tool to evaluate practices in community pharmacies, pharmacists must consider the risks associated with their medicine selection and determine the best practices for risk management specific to their pharmacy.

### 4.3 Strengths and weaknesses of the study

The Delphi consensus method was well suited as a research method for this study. A representative group of medication safety experts from various operating environments was working on the medication safety self-assessment tool for high-alert medications. A key advantage of the method was anonymity, which enabled respondents to freely express their thoughts and take a stand, compared to non-anonymous processes.^42^ Nor was the status of the panel's experts able to influence the opinions of other panellists or the interpretation of the research group.

A further strength of the method is also seen in the fact that in the first Delphi round the experts´ responses were not visible to each other, allowing for unbiased opinions. In the second round, experts could see each other's comments both from the first and the second round in real time, fostering dialogue and enabling comprehensive development of assessment items. The decision to conduct two Delphi rounds was deemed effective, with all evaluation items being assessed in terms of applicability and desirability, and sufficient comments received in each round to reach consensus or carry out necessary modifications.

A significant strength of the Delphi method was the involvement of a large, multi-professional group of experts working in different operating environments, representing participants from all invited professional groups: medical; nursing; and pharmacy professionals, thereby enhancing the reliability of the study. The invited experts were working across Finland. Half of them participated in the first round and one third in the second round. The number of participants was considered sufficient in both Delphi rounds.^39^ Additionally, the participants were actively engaged, and a substantial number of comments were received, with no decrease in quantity toward the end of the tool. The strength of the modification phases lay in having two main researchers, both community pharmacists. Initially, items were examined independently by each researcher, followed by a consensus being reached for each change. If required, the rest of the research team was also consulted.

Most of the assessment items in the first version of the tool were based on ISMP Medication Safety Self Assessment for High-Alert Medications. This was considered a strength of the study, as ISMP has extensive experience in developing medication safety and self-assessment metrics based on actual medication deviations.^38^ The high-alert medications selected for the final tool are also in line with the National Risk Medicines Classification.^7^ However, from the perspective of community pharmacies the original tool was deficient as it lacked several high-alert medication groups commonly found in community pharmacies. These medicines and medicine groups were added to the tool by the research team, based on reliable sources.^7^^,^^47^^,^^48^ The experts provided positive feedback on the new items, with most of them being included in the final tool.

Following modifications based on the comments from the first Delphi round, the evaluation items underwent fewer amendments in the second Delphi round, leading to a consensus on the desirability of the final tool. A consensus rate of 70 % was considered an optimal and achievable limit in this study. However, deviations from this were necessary, for example, to remove duplications. Similarly, some items that did not achieve at least 70 % consensus on applicability or desirability for the tool were included if the research team unanimously deemed the content of the item to be important and desirable.

Some criticism from experts was received during the pilot phase and the first Delphi round, mainly concerning the workload of the evaluation due to the extensive scope of the self-assessment tool. Participation activity decreased in both Delphi rounds toward the end of the tool, particularly weaker in the second round than in the first. The response time of five weeks was deemed too long, since more than 80 % of the responses were received during the last week. In terms of validity, it is essential to consider whether the questions posed were adequately addressed. Experts were asked to evaluate certain items from the perspective of the near future, with a time span of approximately t 10 years. However, these forward-looking development items presented in the tool received criticism from the respondents, and the future was not envisioned much in the comments. The development items of the tool were deemed challenging to evaluate, as not all described operating methods or software-related protections are currently in use in Finnish community pharmacies.

Most of the assessed items received criticism and comments in the first round concerning their content, comprehensibility, clarity, and practical applicability in pharmacy settings. The items were subsequently revised and, in some cases, combined. This also reduced their overall number without compromising valuable content. The reduction in the number of items enhances the tool's usability by ensuring that the self-assessment process remains practical and not overly burdensome for community pharmacies.

### 4.4 Implementation and use of the self-assessment tool

The final self-assessment tool for high-alert medications in Finnish community pharmacies supports the safe use of these medicines and helps pharmacies effectively identify and manage risks. It provides a structured framework for evaluating various aspects of medication safety, enabling pharmacies to develop systematic measures and safeguards. While the self-assessment can be used independently, it is also designed to complement other medication safety evaluation tools, such as the existing general medication safety self-assessment tool for Finnish community pharmacies, and to be integrated into a broader auditing process assessing overall pharmacy medication safety.^36^

In addition to identifying risks, the tool provides practical guidelines and best practices for managing high-alert medications, assisting pharmacies in developing and implementing procedures to minimize associated risks. It includes most commonly used and dispensed high-alert medications in community pharmacies, such as opioids, insulin, NSAIDs, paracetamol. By conducting a self-assessment with the tool, pharmacists can identify areas for improvement in their practices, thereby supporting continuous enhancement of medication safety.

High-alert medications tend to be similar across various countries, including those in Europe, such as Finland and Denmark, as well as in regions like Australia and the USA.^9^^,^^49^^,^^50^ These medications are typically divided into groups that remain consistent across these regions, which makes the self-assessment tool a valuable source of best practices for medication safety worldwide. While minor adjustments can be made to reflect national variations in medication classes, the tool can still be widely used as a reliable, high-quality resource for improving medication safety practice in community pharmacies worldwide. However, it is important to note that the tool's practical use may vary depending on the type of pharmacy, its size, the range of medicines, and its location. For instance, small rural pharmacies may face different challenges and might have limited capacity to implement safeguards for managing high-alert medications, particularly regarding staff, technological support, and access to training By helping to better identify high-alert medications and develop measurements to ensure their safe distribution, particularly through community pharmacies, this tool supports improved safety practices throughout the organization and therefore improved patient safety among pharmacy customers.

### 4.5 Further research

The introduction of the self-assessment tool requires piloting it in pharmacies to gather information on its practical applicability. The tool should be continuously maintained to address emerging challenges related to the safety and use of high-alert medications. When updating the tool, data on reported high-alert medication errors, pharmacies feedback, the National Risk Medicines Classification, and any updated versions of the tool published by ISMP can be utilised.^9^^,^^38^

## 5 Conclusion

The study has demonstrated that it is feasible to develop a self-assessment tool for high-alert medications for the needs of community pharmacies through extensive modification of the original hospital-based ISMP self-assessment tool for high-alert medications. The ISMP tool contained significantly more items for assessment than the final self-assessment tool intended for use by pharmacies. Many of the items in the original tool were related to hospital practices and medications dispensed primarily within hospitals. Additionally, ISMP's tool lacked common high-alert medications found in community pharmacies. The study developed a comprehensive self-assessment tool aimed at promoting the medication safety of high-alert medications in pharmacies. The self-assessment tool is suitable for identifying high-alert medicines, evaluating operating methods and developing protections related to high-alert medicines. The tool could be used to gather information about the challenges and development needs related to high-alert medications in community pharmacies at the national level.

## CRediT authorship contribution statement

**Rositsa Koleva:** Writing – review & editing, Writing – original draft, Visualization, Validation, Software, Resources, Project administration, Methodology, Investigation, Formal analysis, Data curation, Conceptualization. **Anita Währn:** Writing – review & editing, Project administration, Methodology, Investigation, Formal analysis, Data curation, Conceptualization. **Ercan Celikkayalar:** Writing – review & editing, Validation, Supervision, Methodology, Conceptualization. **Sonja Kallio:** Writing – review & editing, Methodology, Conceptualization. **Raisa Laaksonen:** Writing – review & editing, Validation, Supervision, Resources, Project administration, Methodology, Conceptualization.

## Funding

This research did not receive any specific grant from funding agencies in the public, commercial, or not-for-profit sectors. Open access funded by Helsinki University Library.

## Declaration of competing interest

The authors declare that they have no known competing financial interests or personal relationships that could have appeared to influence the work reported in this paper.
